# The effects of c-Src kinase on EMT signaling pathway in human lens epithelial cells associated with lens diseases

**DOI:** 10.1186/s12886-019-1229-4

**Published:** 2019-11-08

**Authors:** Xingyu Li, Fang Wang, Meixia Ren, Minjuan Du, Jian Zhou

**Affiliations:** Department of Ophthalmology, Xijing Hospital, Fourth Military Medical University, Xi’an, 710032 Shaanxi China

**Keywords:** c-Src kinase, Lens epithelial cells, Epithelial to mesenchymal transition, Cataract, Fibrosis

## Abstract

**Background:**

The signaling pathway of epithelial to mesenchymal transition (EMT) is regulated by c-Src kinase in many cells. The purpose of this study was to investigate the effects of c-Src kinase on EMT of human lens epithelial cells in vivo stimulated by different factors.

**Methods:**

Human lens epithelial cells, HLE-B3, were exposed to either an inflammatory factor, specifically IL-1α, IL-6, TNF-α or IL-1β, at 10 ng/mL or high glucose (35.5 mM) for 30 mins. Activity of c-Src kinase was evaluated by the expression of p-Src^418^ with western blot assay. To investigate the effects of activation of c-Src on EMT, HLE-B3 cells were transfected with pCDNA3.1-Src^Y530F^ to upregulate activity of c-Src kinase, and pSlience4.1-ShSrc to knock it down. The expressions of c-Src kinase and molecular markers of EMT such as E-cadherin, ZO-1, α-SMA, and Vimentin were examined at 48 h by RT-PCR and western blot. At 48 h and 72 h of transfection, cell proliferation was detected by MTT, and cell mobility and migration were determined by scratch and transwell assays.

**Results:**

Activity of c-Src kinase, which causes the expression of p-Src^418^, was upregulated by different inflammatory factors and high glucose in HLE-B3 cells. When HLE-B3 cells were transfected with pCDNA3.1-Src^Y530F^, the expression of c-Src kinase was upregulated on both mRNA and protein levels, and activity of c-Src kinase, expression of p-Src^418^ increased. The expressions of both E-cadherin and ZO-1 were suppressed, while the expressions of vimentin and α-SMA were elevated on both mRNA and protein levels at the same time. Cell proliferation, mobility and migration increased along with activation of c-Src kinase. Conversely, when HLE-B3 cells were transfected with pSlience4.1-ShSrc, both c-Src kinase and p-Src^418^ expressions were knocked down. The expressions of E-cadherin and ZO-1 increased, but the expressions of Vimentin and α-SMA decreased; meanwhile, cell proliferation, mobility and migration reduced.

**Conclusions:**

The c-Src kinase in lens epithelial cells is easily activated by external stimuli, resulting in the induction of cell proliferation, mobility, migration and EMT.

## Background

Previous studies have shown that lens fibrotic disorders, such as anterior subcapsular cataract (ASC) and posterior capsular opacification (PCO), are common types of cataract and visual impairment. ASC is a primary cataract, which is characterized by dense fibrotic regions underneath the anterior capsule and is mainly caused by inflammation, ocular trauma and irritation [1]. PCO, a secondary cataract, occurs in 30 to 50% of adults and almost 100% of children who receive cataract surgery [2], and it is associated with fibrosis and contraction of the posterior lens capsule [2–4]. ASC and PCO share many molecular features such as aberrant proliferation, migration and epithelial to mesenchymal transition (EMT) of lens epithelial cells (LECs) [5]. Accumulating evidence shows that anti-inflammation treatments after cataract surgery could reduce migration and fibrosis of LECs [6–8]. It has been reported that fibrosis of LECs in patients with diabetes mellitus was significantly higher than in patients without diabetes at 6 and 12 months after cataract extraction [9]. These studies suggest that inflammatory factors and high glucose are the stimulating factors for fibrosis of LECs.

EMT is associated with many molecular and morphologic changes to epithelial cells that enable them to lose their cell polarity and cell-cell adhesion, gain properties in migration and invasion and become mesenchymal cells [10, 11]. The most marked characteristics of EMT are loss of epithelial markers, such as E-cadherin and ZO-1, and acquisition of a spindle shape cell, which is accompanied by accumulation of Vimentin and a-smooth muscle actin (a-SMA) [12]. This specific process is present in embryonic development, wound healing and tissue repairment and tumor metastasis. In organ fibrosis such as renal fibrosis, pulmonary fibrosis, hepatic fibrosis and ocular fibrosis, EMT is triggered by various biomolecules and signaling pathways, such as transforming growth factor-β (TGF-β) [13], insulin-like growth factor-1 (IGF-1) [14], transcription factor snail [15], and PI3K/Akt/mTOR/NF-κB signaling [16].

c-Src kinase, one of the Src-family tyrosine kinases (SFKs), is activated by many stimulators, such as epidermal growth factor receptor (EGFR) [17], P2RY2 (a purinergic GPCR receptor) and reactive oxygen species (ROS) [18], high glucose [19], heterotrimeric G protein-coupled receptors [20], PKA signaling [21] and the pathways of IL-1 and EGFR/integrin signaling [22]. Activation of c-Src kinase is required for cell differentiation, migration and change of intercellular junction, including cadherin-based intercellular adhesions and integrin-mediated cell-matrix adhesions of epithelial cells, particularly during EMT [23, 24]. Inhibition of SFKs with their specific inhibitors attenuates fibrosis in lung, pancreas and skin, which suggests that activation of Src kinase is an attractive trigger point of organ fibrosis [25, 26]. In lens epithelial cells, activation of Src kinase induced by serum increased cell migration, weakened cell-cell junctions, and caused lens epithelial cells to acquire the phenotype of mesenchymal cells [27].

The c-Src kinase is made up of a lipophilic N-terminus, followed by the regulatory SH3 and SH2 domains, a catalytic protein tyrosine kinase (PTK) core, and a c-terminus regulatory tail [28–30]. The PTK domain contains the kinase domain and a conserved tyrosine residue involved in autophosphorylation. Phosphorylation of the Tyr 418 residue of the PTK domain is required for maximum kinase activity [31]. A negative regulatory domain is adjacent to the PTK domain. Phosphorylated Tyr 530 interacts and binds with the SH2 domain to keep the SFK in the inactive conformation. In other words, c-Src kinase is activated by phosphorylation at Tyr 418 or dephosphorylation at Tyr 530 [32].

In the present study, we hypothesized that activation of c-Src kinase stimulated by a variety of factors, such as inflammatory factors or high glucose, could be a trigger for EMT of LECs. By transfecting HLE-B3 cells with c-Src activated vector or ShRNA vector, the effects of c-Src kinase on cell proliferation, mobility, migration and EMT were observed.

## Methods

### Plasmid construction

Construction of pCDNA3.1-c-Src^Y530F^ recombination vector was described previously [33–35]. Mutant c-Src^Y530F^ cDNA generated by RT-PCR from total mRNA of HLE-B3 cells with the primers:

sense 5′-GGCAAGCTTATGGGTAGCAACAAGAGCAAGCCCAAG-3′;

antisense 5′-GCTCTAGACTAGAGGTTCTCCCCGGGCTGGAACTG-3′,

(underlined sequences was the mutation site) was cloned into the expression vector pCDNA3.1 (Invitrogen, Carlsbad, CA, USA), creating pCDNA3.1-c-Src^Y530F^ recombination vector. ShRNA expression vectors were generated by annealing single-stranded oligonucleotides and inserting them into the BamHI and HindIII enzyme sites of pSilencer4.1-CMVneo vector (Ambion, Austin, TX, USA). The target sequences were as follows: ShSrc (c-Src, NM_005417, 1682–1700 bp): 5′-TCGGCTCATTGAAGACAAT-3′ (provided by Genepharma Inc., Shanghai, China), and a scrambled sequence was used as a negative control (ShNC): 5′-TTCTCCGAACGTGTCACGT-3′ (Ambion, Austin, TX, USA). The recombinant ShRNA vectors were named as pSlience4.1-ShSrc and pSlience4.1-ShNC.

### Cell culture

Human lens epithelial cells, HLE-B3 cells were grown adherently in Dulbecco’s modified Eagle’s medium (DMEM, with 5.5 mM glucose) with 10% fetal bovine serum and 2 mM L-glutamine, and incubated at 37 °C with 5% CO_2_ [19]. All cells used in the experiments were taken in logarithmic phase.

### Groups and treatment

Groups of stimulation with inflammatory factors: in the treatment groups, HLE-B3 cells were treated with IL-1α, IL-6, TNF-α, or IL-1β at 10 ng/mL for 30 mins. In the control group, HLE-B3 cells were cultured in DMEM with 0.5% fetal bovine serum for 30 mins.

Groups of stimulation with high glucose: in the control group (5.5 mM), HLE-B3 cells were cultured in DMEM with 5.5 mM glucose in the medium for 30 mins; in the osmotic control group (mannitol), the cultured cells were treated with 30 mM mannitol for 30 mins; and in the high glucose group (35.5 mM), the cells were treated with 30 mM glucose for 30 mins.

Groups of c-Src kinase activation: in the c-Src activation group (pCDNA3.1-c-Src^Y530F^), HLE-B3 cells were transfected with pCDNA3.1-c-Src^Y530F^ recombination vector. In the blank control group (pCDNA3.1), cells were transfected with blank vector, pCDNA3.1 vector. In the negative control group (control), cells were transfected with transfection reagent Lipofectamine 2000.

Groups of c-Src kinase inhibition: in the c-Src silence group (ShSrc), HLE-B3 cells were transfected with pSlience4.1-ShSrc vector. In the blank control group (ShNC), the cells were transfected with pSlience4.1-ShNC vector, and in the negative control group (control), cells were transfected with transfection reagent Lipofectamine 2000.

### Transfection

HLE-B3 cells (2 × 10^5^ cells/well) were seeded in 6-well plates and grown overnight to 80% confluence prior to transfection. All transfections for plasmids were performed with Lipofectamine 2000 (Invitrogen, Carlsbad, CA, USA) according to the manufacturer’s instructions. Plasmid DNA-lipid complexes (4 μg plasmids in 8 μl Lipofectamine 2000 per well) were quickly prepared and then incubated for 20 mins at room temperature. The DNA-lipid complexes were added to HLE-B3 cells in 6-well plates and were cultured for 4–6 h. Lastly, DNA-lipid complexes were discarded, and 2 ml complete medium were added. Finally, 400 μg G418 (Invitrogen, Carlsbad, CA, USA) was applied to select neomycin-resistant cells.

### Quantitative real-time PCR (RT-PCR)

Total RNA was extracted from cells using Trizol reagent (Takara, Dalian, China), and 1 μg total RNA was used as the template for cDNA synthesis in a reverse transcription kit (Takara, Dalian, China). RT-PCR was performed in the SYBR Green kit (Takara, Dalian, China) using specific primers for c-Src, E-Cadherin, ZO-1, Vimentin and α-SMA (Table 1). The relative expression levels of genes were normalized to the endogenous housekeeping gene GAPDH.

**Table 1:** Primers for quantitative real-time PCR

Gene	GenBank ID	Primer pairs (5′ → 3′)
c-Src	NM_005417	F:AAGCCTGGCACGATGTCT R:CGATGTAAATGGGCTCCTCT
E-Cadherin	AB025106	F:CCCCGCCTTATGATTCTC R: GCCCCATTCGTTCAAGTA
ZO-1	NM_003257	F: CTGCTTGACCTCCCTAAA R: ATCCACAACACGGAACAC
Vimentin	NM_003380	F:AGCTCCAGCCGGAGCTAC R: CGCTGCTCCGCAGGCGCA
α-SMA	NM_001141945	F: TGCTCCCAGGGCTGTTTT R: GCCATGTTCTATCGGGTACTTC
GAPDH	NG 007073.2	F: CCACATCGCTCAGACACCAT R:GGCAACAATATCCACTTTACCAGAGT

### Western blot

Cells were harvested and lysed in RIRA cell lysate (50 mM Tris (pH 7.4), 150 mM NaCl, 1% Triton X-100, 1% sodium deoxycholate, 0.1% SDS and 0.5 mM PMSF) (Beyotime Biotechnology, Beijing, China), and total proteins were extracted. The proteins were quantified by BCA assay kit (Beyotime Biotechnology, Beijing, China), and 5 × loading buffer (250 mM Tris-HCl (pH 6.8), 10% SDS, 0.5% BPB, 50% Glycerin, 5% 2-Mercaptoethanol) was added to the proteins. Next, 35 μg total proteins from each sample were uploaded and separated in 12% SDS-PAGE at 120 V voltage, then transferred onto PVDF membranes at 40 mA for 2.5 h at room temperature. According to the standard protein marker, the PVDF membrane was cut according to the molecular weight of the target protein. After blocking by 5% defatted milk powder for 2 h at room temperature, PVDF membrane bands were incubated with primary antibodies overnight at 4 °C, such as, c-Src (1:1000, ab109381, Abcam, Cambridge, UK), p-Src^418^ (1:500, ab133460, Abcam, Cambridge, UK), E-Cadherin (1:200, #3195, Cell Signal Technology, Danvers, MA, USA), ZO-1 (1:1000, 21,773–1-AP, Proteintech, Chicago, IL, USA), Vimentin (1:1000, ab92547, Abcam, Cambridge, UK), α-SMA (1:1000, ab124964, Abcam, Cambridge, UK) and GAPDH (1:2000, Cell Signal Technology, Danvers, MA, USA). After being washed with TBST three times (for 15 mins each) the next day, PVDF membrane bands were incubated with secondary antibodies (1:4000, Cell Signal Technology, Danvers, MA, USA) for 4 hrs at 4 °C. After being washed with TBST, proteins in PVDF membrane bands were detected with luminol reagent (Santa Cruz, USA). GAPDH expression was used as the internal standard. The protein bands were observed and captured with a scanner (HP Deskjet F2288, China). The images were analyzed by Quantity One software.

### Cell proliferation assay

Cell proliferation was evaluated by MTT (Sigma, St Louis, MO, USA) assay at indicated time points [36]. HLE-B3 cells (2 × 10^5^ cells/well) were seeded in 6-well plates. The next day, cells were transfected with pCDNA3.1-c-Src^Y530F^, pSlience4.1-ShSrc and control vectors. After transfection for 24 h, cells in activation of c-Src kinase group, inhibition of c-Src kinase group and control group were trypsinized and seeded in 96-well plates (6 × 10^3^ cells/well). At different time points (after sticking for 0, 12, 24, 48 and 72 h), the medium was replaced with 100 μl MTT (5 mg/ml), and the plate was incubated at 37 °C for another 4 h. After incubation, the culture medium was removed gently, and 100 μl DMSO was added. Finally, the absorbance was determined on a microreader (Bio-Rad, Hercules, CA, USA) at 570 nm. All experiments were performed three times independently. The cell proliferation diagram was plotted using the absorbance at each time point.

### Scratch assay

HLE-B3 cells (2 × 10^5^ cells/well) were seeded in 6-well plates. On the second day, cells were transfected with pCDNA3.1-c-Src^Y530F^, pSlience4.1-ShSrc and control vectors. After 24 h, cells in each well were scratched with a 200 μl pipette tip. Once scratches were made, the plates were gently washed with PBS three times, and then 2 ml serum-free medium was added. Cell mobility was examined after 24 h and 48 h. The images at 0 h (T0), which was just after scratching, 24 h (T24) and 48 h (T48) were taken with a digital camera (Olympus DP71, Japan) connected to an inverted microscope (Olympus IX71, Japan). Ten fields of each plate were picked randomly and marked. Measurements of the width of gap were repeated three times at the same field. Gap closure (%) = [Gap in width (T0- T24/48)/Gap in width T0] × 100%.

### Transwell assay

Cell migration was determined using transwell assay (Corning incorporated, NY, USA). HLE-B3 cells (2 × 10^5^ cells/well) were seeded in 6-well plates. On the following day, cells were transfected with pCDNA3.1-c-Src^Y530F^, pSlience4.1-ShSrc vector and control vectors. After transfection for 24 h, cells in each group were trypsinized and seeded in matrigel coated filters (2 × 10^4^ cells/well) and cultured with 100 μl serum-free medium. Then 600 μl completed medium was added into the lower compartment of chamber. After incubation for 24 h and 48 h, cells on the upper surface of the filter were wiped off with a swab, whereas cells that had passed through the filter were fixed with 95% ethanol, stained with crystal violet and counted under the microscope. Relative migration was based on the average number of cells on the underside of the membrane in 10 random images generated at 4× magnification under the microscope.

### Data analysis and statistics

Each experiment was repeated three times independently, and all results were presented as mean ± standard deviation (SD). All data were analyzed using SPSS 19.0 software. Multiple-group comparison was performed by analysis of variance (ANOVA), followed by LSD test for between-group comparison. Values of *P* < 0.05 were considered as significant and indicated by asterisks in the figures.

## Results

### Inflammatory factors and high glucose activated c-Src kinase

**Fig. 1:**
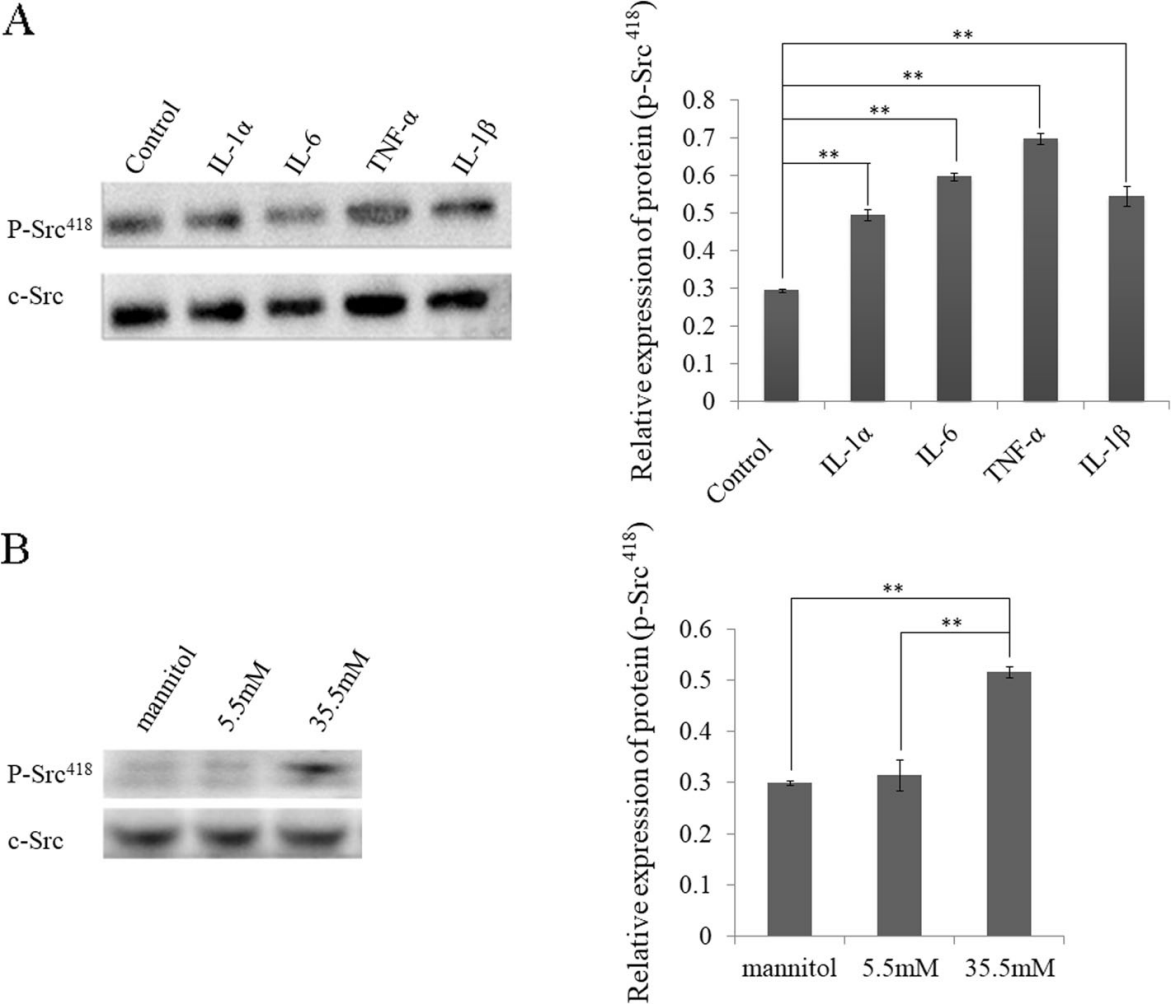
The c-Src kinase was activated by inflammatory factors and high glucose **a** Left, the expressions of c-Src and p-Src^418^ (active c-Src kinase) were examined by western blot at 30 mins after treatment with inflammatory factors in HLE-B3 cells. Right, the relative expression of p-Src^418^, radio of p-Src^418^/c-Src. **b** Left, the expressions of c-Src and p-Src^418^ were examined by western blot in different concentrations of glucose. Right, the relative expression of p-Src^418^, radio of p-Src^418^/c-Src. (**P < 0.01 compared with control, *n* = 3)

Using western blot assay, we found that after treatment with inflammatory factors IL-1α, IL-6, TNF-α and IL-1β for 30 mins, the activity of c-Src kinase (gray ratio of p-Src^418^/c-Src) in HLE-B3 cells was enhanced significantly compared with the control group. The effect of TNF-α on activation of c-Src kinase was the strongest (Fig. 1a). The expression of p-Src^418^ in the 35.5 mM glucose group was significantly higher than that in the 5.5 mM glucose group and mannitol group, whereas that in the mannitol group was almost the same as in the 5.5 mM glucose group (Fig. 1b). These results suggested that both inflammatory factors and high glucose stimulated the activity of c-Src kinase in HLE-B3 cells.

### Alteration of c-Src kinase activity in HLE-B3 transfected with pCDNA3.1-c-Src^Y530F^ vector or pSlience4.1-ShSrc vector

**Fig. 2:**
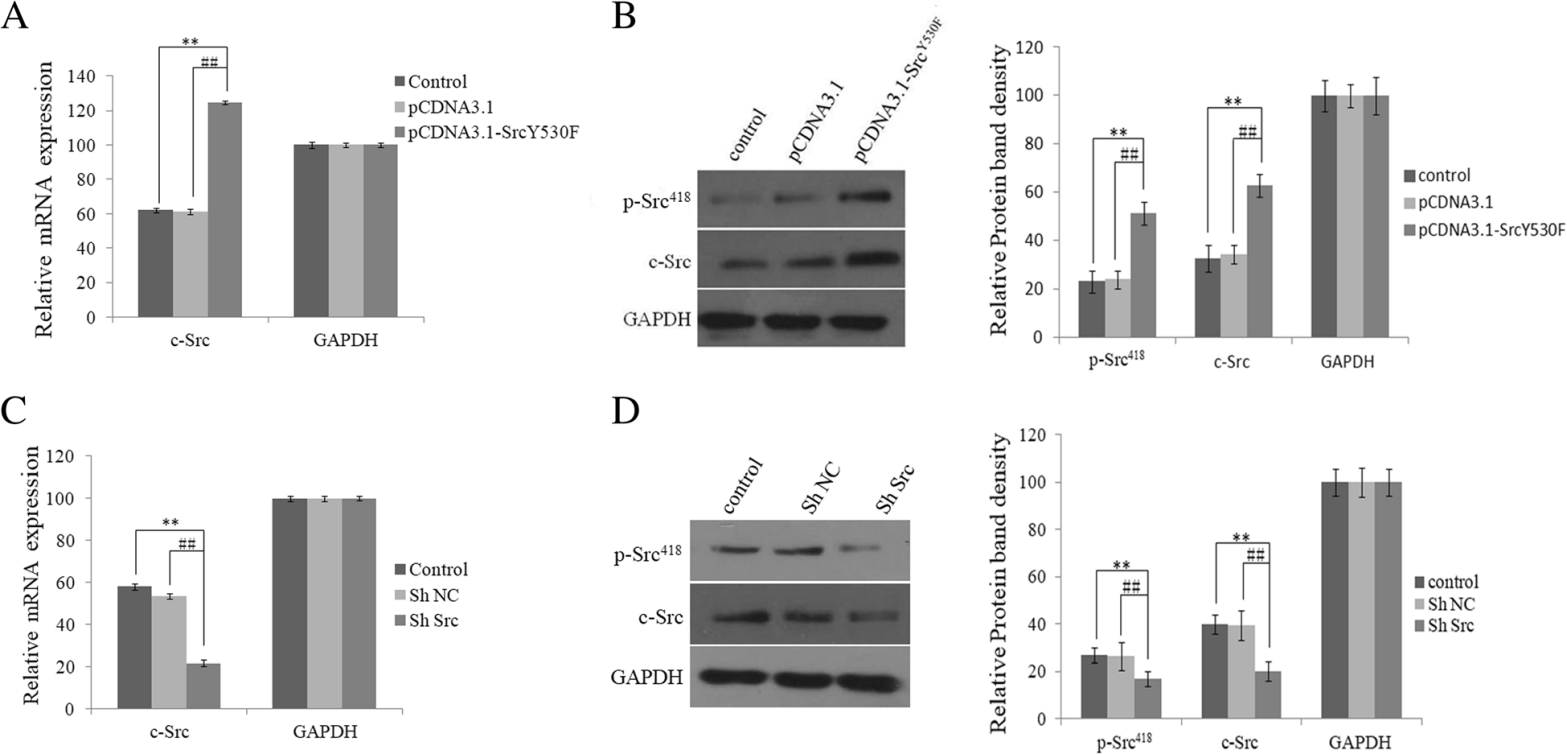
Alterations of c-Src kinase in HLE-B3 transfected with pCDNA3.1-c-Src^Y530F^ vector or pSlience4.1-ShSrc vector **a** The expressions of mRNA of c-Src kinase examined by RT-PCR in HLE-B3 cells transfected with pCDNA3.1-c-Src^Y530F^ vector (group pCDNA3.1-c-Src^Y530F^), pCDNA3.1 vector (group pCDNA3.1), and Lipofectamine 2000 (group control) at 48 h. **b** Left, the expressions of c-Src kinase examined by western blot at 48 h after transfection. Right, relative expression of protein in left figure. (*P < 0.05, **P < 0.01 compared with control, #*P* < 0.05, ##*P* < 0.01 compared with group pCDNA3.1, *n* = 3). **c** The expression of c-Src was examined by RT-PCR in HLE-B3 cells transfected with pSlience4.1-ShSrc vector (group ShSrc), pSlience4.1-ShSrc (group ShNC), and Lipofectamine 2000 (group control) at 48 h after transfection. **d** Left, the expressions of c-Src kinase (c-Src) and activated c-Src kinase (p-Src 418) examined by western blot at 48 h after transfection. Right, relative expression of protein in left figure. (*P < 0.05, **P < 0.01 compared with control, #*P* < 0.05, ##*P* < 0.01 compared with group ShNC, *n* = 3)

First, pCDNA3.1-c-Src^Y530F^ and pSlience4.1-ShSrc vectors were constructed and transfected into HLE-B3 cells for 48 h. To evaluate the activating and inhibitive effects on c-Src kinase, RT-PCR and western blot assay were applied to examine the expressions of c-Src kinase on mRNA and protein levels, respectively. In HLE-B3 cells transfected with pCDNA3.1-c-Src^Y530F^ (group pCDNA3.1-c-Src^Y530F^), the expressions of c-Src mRNA and protein were significantly higher than two control groups (group pCDNA3.1 and group control). Furthermore, activity of c-Src kinase, the expression of p-Src^418^, was much higher than controls (Fig. 2a and b), suggesting that c-Src kinase was activated by pCDNA3.1-Src^Y530F^ vectors in the cells. In HLE-B3 cells transfected with pSlience4.1-ShSrc vector (group ShSrc), obvious suppressions of c-Src in mRNA and protein levels were demonstrated, and p-Src^418^ protein expression was also decreased (Fig. 2c and d), implying that expression of endogenous c-Src was suppressed by ShRNA.

### Activation of c-Src kinase promoted EMT of HLE-B3 cells

**Fig. 3:**
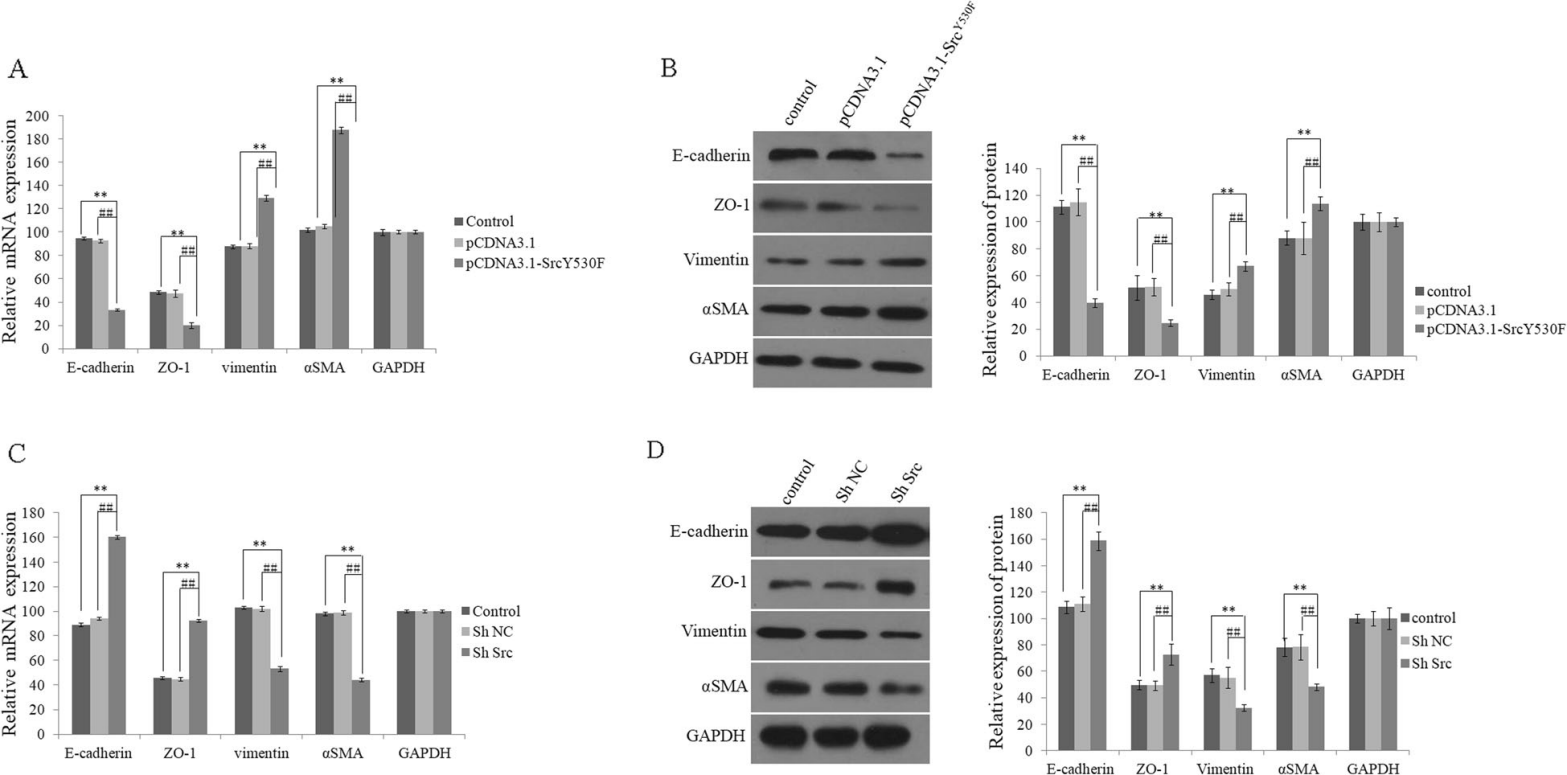
Effects of c-Src on the expressions of EMT marker proteins in HLE-B3 cells **a** The expressions of EMT marker proteins were examined by RT-PCR in group pCDNA3.1-c-Src^Y530F^, group pCDNA3.1 and group control at 48 h after transfection. **b** Left, the expressions of EMT marker proteins were examined by western blot in group pCDNA3.1-c-Src^Y530F^, group pCDNA3.1 and group control at 48 h after transfection. Right, relative expression of protein in left figure. (*P < 0.05, **P < 0.01 compared with control, #*P* < 0.05, ##*P* < 0.01 compared with group pCDNA3.1, *n* = 3). **c** The expression of EMT marker proteins was examined by RT-PCR in group ShSrc, group ShNC and group control at 48 h after transfection. **d** Left, the expressions of EMT marker proteins were examined by western blot in group ShSrc, group ShNC and group control at 48 h after transfection. Right, relative expression of protein in left figure. (*P < 0.05, **P < 0.01 compared with control, #*P* < 0.05, ##*P* < 0.01 compared with group ShNC, *n* = 3)

To explore the biological roles of activation of c-Src kinase on EMT of LECs, we further examined the expression of epithelial cell proteins such as E-cadherin and ZO-1, and the mesenchymal cell proteins such as Vimentin and α-SMA in cells of group pCDNA3.1-c-Src^Y530F^, in which c-Src kinase was activated. It was shown by RT-PCR and western blot that the expression of both E-cadherin and ZO-1 reduced significantly, and Vimentin and α-SMA increased dramatically compared with cells in control groups (group pCDNA3.1 and group control) (Fig. 3a and b). In pSlience4.1-ShSrc vector transfected cells, in which c-Src was knocked down, the expressions of E-cadherin and ZO-1 increased, while Vimentin and α-SMA reduced significantly compared with control cells (group ShNC and group control) (Fig. 3c and d). Altogether, activation of c-Src kinase could induce EMT process in HLE-B3 cells.

### Activation of c-Src kinase stimulated cell proliferation

**Fig. 4:**
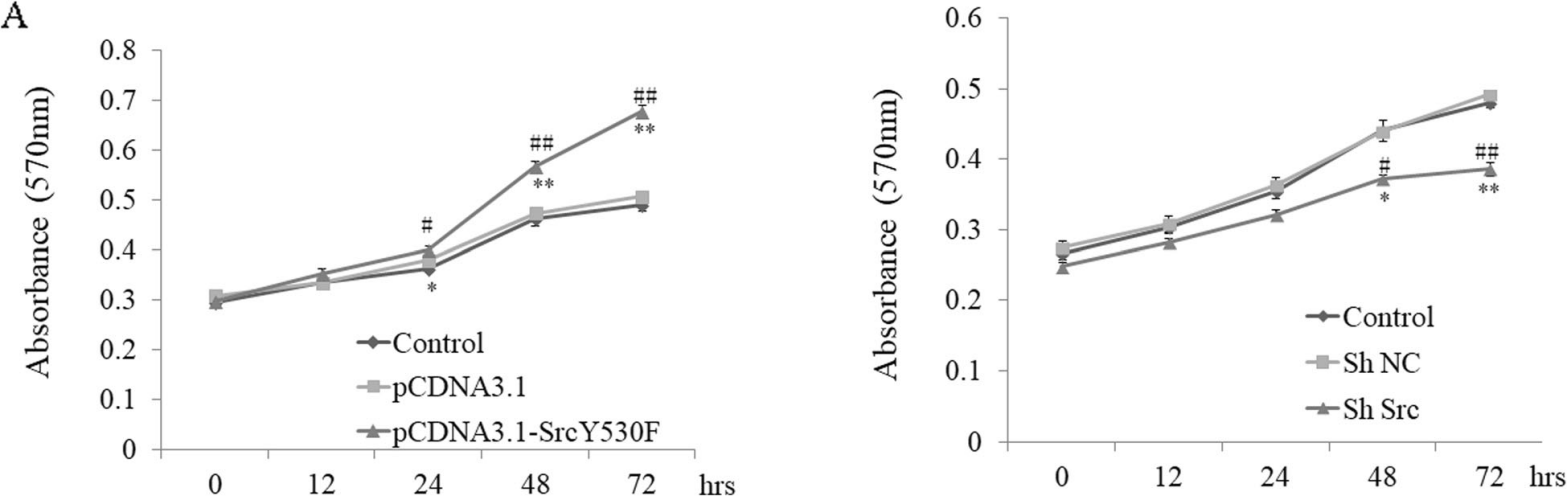
Effect of c-Src on cell proliferation by MTT assay **a** The effect of c-Src on cell proliferation examined by MTT assay in group pCDNA3.1-c-Src^Y530F^, group pCDNA3.1 and group control. (*P < 0.05, **P < 0.01 compared with control, #*P* < 0.05, ##*P* < 0.01 compared with group pCDNA3.1, *n* = 3). **b** The effect of c-Src on cell proliferation examined by MTT assay in group ShSrc, group ShNC and group control. (*P < 0.05, **P < 0.01 compared with control, #*P* < 0.05, ##*P* < 0.01 compared with group ShNC, *n* = 3)

MTT assay showed that proliferation of cells in group pCDNA3.1-c-Src^Y530F^ increased by 9, 9, 25 and 39% compared with that in group pCDNA3.1 at 12 h, 24 h, 48 h and 72 h, respectively. The proliferation of cells in group pCDNA3.1- c-Src^Y530F^ increased by 4, 9, 21 and 37% compared with that in the control group at 12 h, 24 h, 48 h and 72 h, respectively (Fig. 4a), while in cells of group ShSrc, the proliferation did not change at 12 h compared with two control groups, but reduced by 2, 7 and 13% compared to group ShNC, and 3, 8 and 14% compared to control group at 24 h, 48 h and 72 h, respectively (Fig. 4b). This indicated that activation of c-Src kinase stimulated cell proliferation.

### Activation of c-Src kinase increased cell mobility and migration

**Fig. 5:**
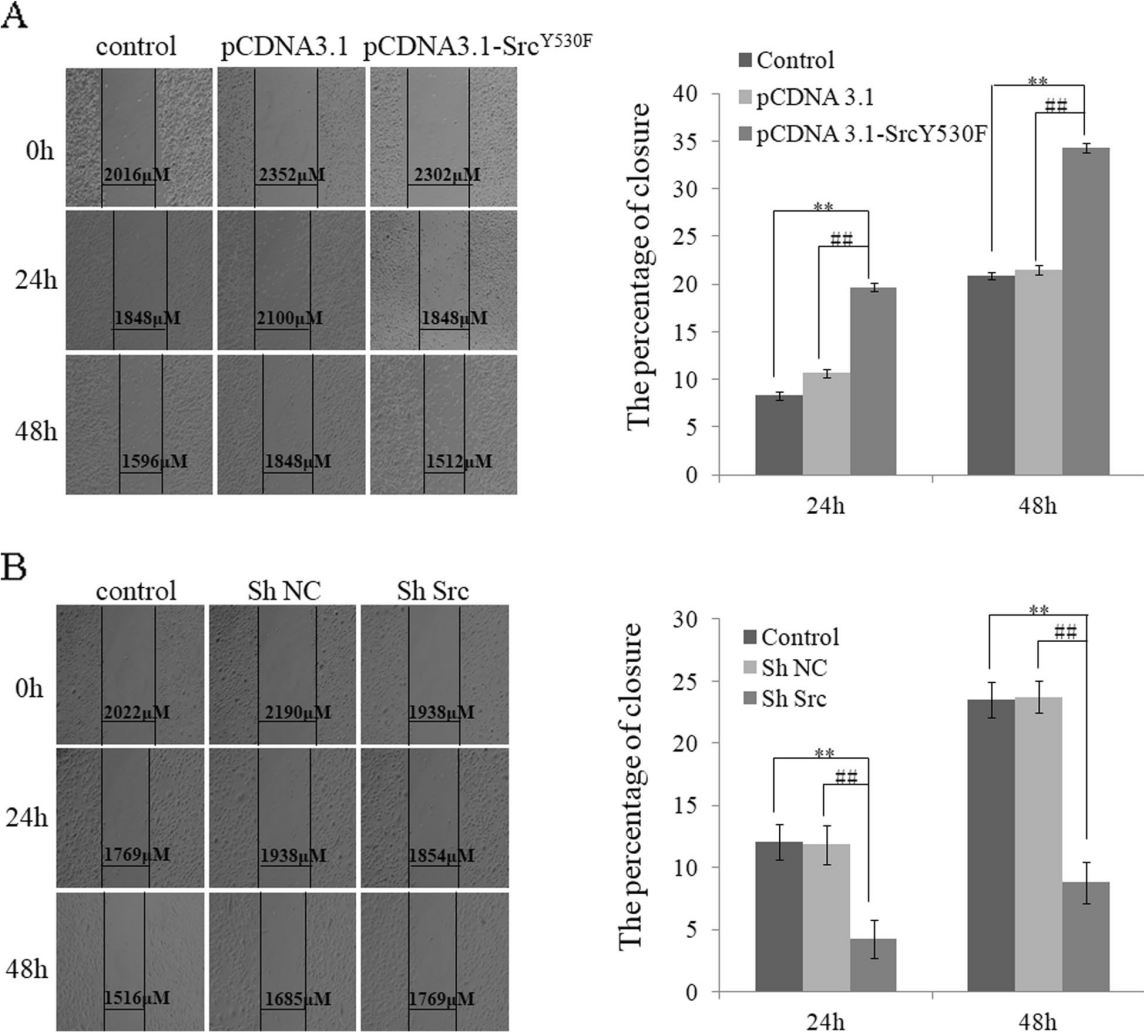
Effect of c-Src on cell migration **a** The effect of c-Src on cell migration examined by scratch assay in group pCDNA3.1-c-Src^Y530F^, group pCDNA3.1 and control. (*P < 0.05, **P < 0.01 compared with control, #*P* < 0.05, ##*P* < 0.01 compared with group pCDNA3.1, *n* = 3). **b** The effect of c-Src on cell migration examined by scratch assay in group ShSrc, group ShNC and control. (*P < 0.05, **P < 0.01 compared with control, #*P* < 0.05, ##*P* < 0.01 compared with group ShNC, *n* = 3)

By scratch assay, gap closure in cells of group pCDNA3.1- c-Src^Y530F^ increased by 84, 60% compared with group pCDNA3.1 and 137, 65% compared with the control group at 24 h and 48 h, respectively, which suggested the enhancing of migration ability after activation of c-Src kinase in HLE-B3 cells (Fig. 5a), while in HLE-B3 cells transfected with pSlience4.1-ShSrc vector (group ShRNA), gap closure was reduced by 63, 62% compared with group ShNC and 65, 65% compared with control group at 24 h and 48 h, respectively (Fig. 5b).

**Fig. 6:**
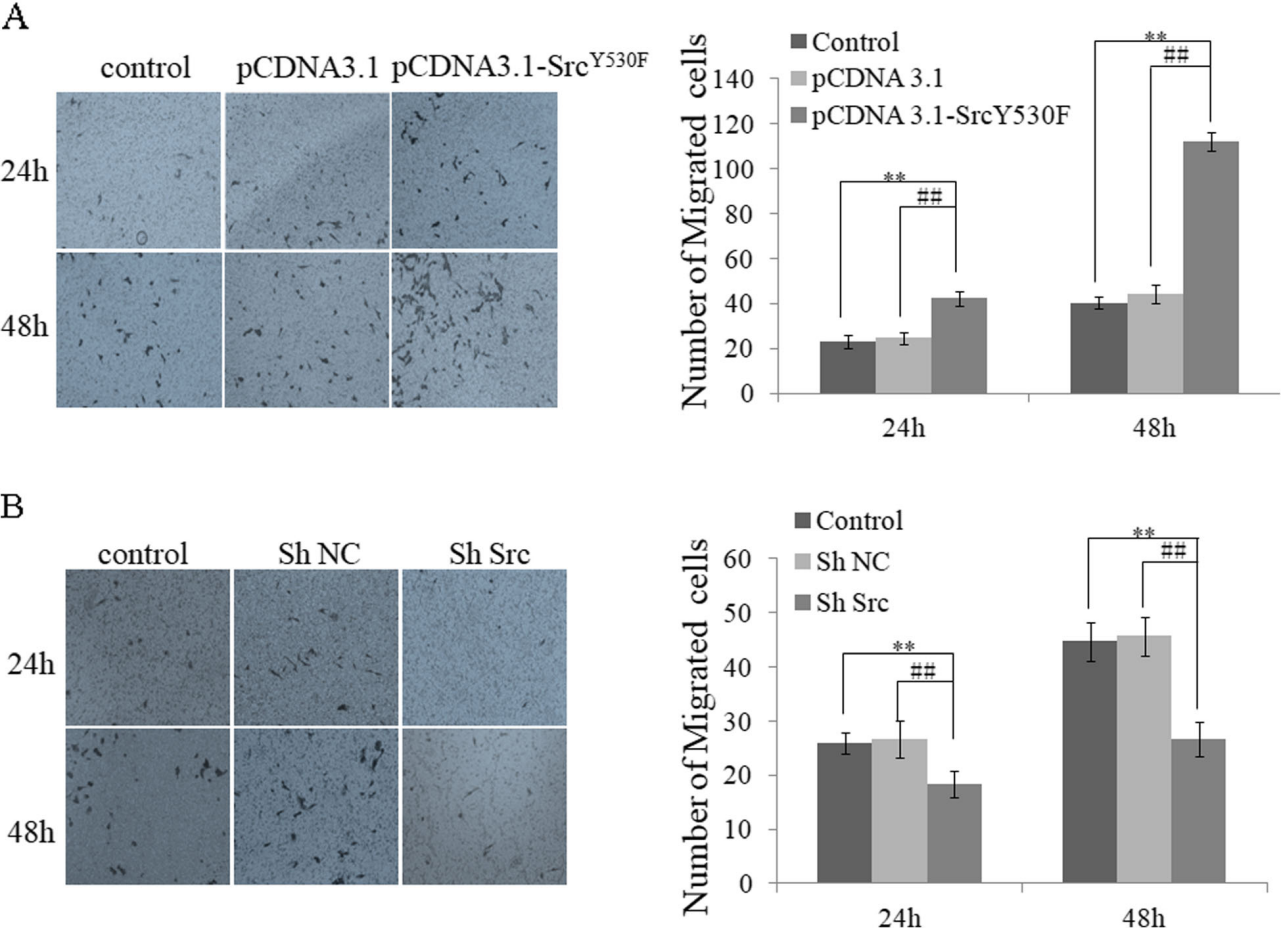
Effect of c-Src on cell mobility **a** The effect of c-Src on cell mobility examined by transwell assay in group pCDNA3.1-c-Src^Y530F^, group pCDNA3.1 and control. (*P < 0.05, **P < 0.01 compared with control, #*P* < 0.05, ##*P* < 0.01 compared with group pCDNA3.1, *n* = 3). **b** The effect of c-Src on cell mobility examined by transwell assay in group ShSrc, group ShNC and control. (*P < 0.05, **P < 0.01 compared with control, #*P* < 0.05, ##*P* < 0.01 compared with group ShNC, *n* = 3)

In transwell assay, the migrating cell number in group pCDNA3.1- c-Src^Y530F^ increased by 71, 152.8% compared with group pCDNA3.1, and 83.9, 177.9% compared with the control group at 24 h and 48 h, respectively (Fig. 6a). In contrast, in cells of group ShSrc, this number was reduced by 31.5, 41.6% compared to group ShNC, and 29.6, 40.3% compared with control group at 24 h and 48 h, respectively (Fig. 6b). This suggested that activation of c-Src could induce cell mobility and migration.

## Discussion

EMT is a conserved and essential process shared by developmental morphogenesis and carcinogenesis as well as physiological response to injury, operations and tissue fibrotic diseases. In lens fibrotic diseases, EMT is an important pathological process; for instance, in ASC and PCO. Our present study showed that inflammatory factors and high glucose stimulated the activity of c-Src kinase in HLE-B3 cells, and activation of c-Src kinase promoted the EMT process, cell migration and cell proliferation in HLE-B3 cells. In contrast, when c-Src kinase was inhibited, the EMT process and cell migration and proliferation were reduced. All of these data suggested that c-Src is a key regulator in the lens diseases associated with EMT.

Activation of c-Src kinase could promote the EMT process as well as cell migration and proliferation in HLE-B3 cells, which is consistent with the role of c-Src kinase in tumors. Activation of c-Src kinase affects the EMT process and enhances cell migration and proliferation in many cancer cells [37, 38]. In various types of cancer, such as breast cancer, pancreatic cancer and castration-resistant prostate cancer, activation of c-Src increases cell invasiveness by altering the activity of cadherins, adhesion proteins and integrins [39].

Src activation stimulates downstream kinases such as extracellular signal-regulated kinase (ERK) [40] and GSK3 [41, 42], which are involved in the regulation of cell survival and proliferation and the promotion of EMT. The interaction of activated Src with p120-catenin may cause dissociation of cell-cell junctions, thus facilitating cell mobility [43]. Similarly, LECs in the EMT process experience a loss of cell adhesion from epithelial cells and gain the ability of proliferation and migration as they transition into mesenchymal cells [44]. In fibroblasts, the binding of integrins to their ligands leads to activation of focal adhesion plaques adhesion kinase (FAK), which, in turn, recruits and activates c-Src [45]. Furthermore, activation of c-Src is required to disrupt cadherin-dependent cell-cell contact [46].

Our results showed that activation of c-Src reduced the expression of E-cadherin at the protein and mRNA levels in HLE-B3 cells. This may be one of the mechanisms by which activation of c-Src induces EMT of lens epithelial cells. E-cadherin is the major cadherin molecule expressed in epithelial cells and is down-regulated in mesenchymal cells [47]. Loss of E-cadherin is the characteristic associated with the increasing potential to invade surrounding tissues and disseminate to distant sites, and it is the hallmark of EMT [48–50]. E-cadherin is a single-span transmembrane glycoprotein that maintains intercellular contacts and cellular polarity in epithelial tissues. In tumor cells, loss of E-cadherin is associated with cell invasion and metastasis [51]. In pancreatic ductal adenocarcinoma (PDAC) cell lines, overexpression of activated c-Src induces down-regulation of E-cadherin [52]. When c-Src binds to E-cadherin, it disrupts cell-cell interaction, enabling cancer cells to detach from their original site [53]. These results from previous studies supported our findings that activation of c-Src kinase increased cell motility and induced EMT.

It is known that TGF-β plays a key role in EMT of epithelial cells through TGF-β/Smad [54, 55] and TGF-β/HIF-1α signaling pathways [56]. Our previous study suggested that c-Src kinase regulated EMT of LECs through TGF-β signaling pathway, based on the evidence that following the inhibition of c-Src kinase in LECs stimulated by high glucose, the expression of TGF-β and its receptor ALK5 decreased significantly, and EMT of HLE-B3 cells was prevented [19].

## Conclusions

The results of this study indicated that c-Src kinase could be activated by inflammatory factors and high glucose. Activation of c-Src kinase elevated EMT and cell proliferation, mobility and migration of LECs, whereas silencing the c-Src gene in HLE-B3 led to the opposite effects. Activation of c-Src was a trigger for EMT of lens epithelial cells associated with fibrosis of lens diseases.

## Data Availability

All data generated or analyzed during this study are included in this published article. More details are available from the corresponding author on reasonable request.

## Abbreviations

ASC: Anterior subcapsular cataract
a-SMA: a-smooth muscle actin
DMEM: Dulbecco’s modified Eagle’s medium
EGFR: Epidermal growth factor receptor
EMT: Epithelial-mesenchymal transition
ERK: Extracellular signal-regulated kinase
FAK: Focal adhesion plaques adhesion kinase
IGF-1: Insulin-like grow factor-1
LECs: Lens epithelial cells
PCO: Posterior capsular opacification
PDAC: Pancreatic ductal adenocarcinoma
PTK: Protein tyrosine kinase
ROS: Reactive oxygen species
SFKs: Src- family tyrosine kinases
TGF-β: Transforming growth factor-β
